# Complexities of gender assignment in 17β-hydroxysteroid dehydrogenase type 3 deficiency: is there a role for early orchiectomy?

**DOI:** 10.1186/1687-9856-2013-15

**Published:** 2013-09-12

**Authors:** Janet Chuang, Amy Vallerie, Lesley Breech, Howard M Saal, Shumyle Alam, Peggy Crawford, Meilan M Rutter

**Affiliations:** 1 Division of Endocrinology, Cincinnati Children's Hospital Medical Center, 3333 Burnet Avenue, Cincinnati, Ohio 45229, USA; 2 Adolescent Medicine, New York Medical College, Munger Pavilion, Rm 123, Valhalla, New York 10595, USA; 3 Pediatric and Adolescent Gynecology, Cincinnati Children’s Hospital Medical Center, 3333 Burnet Avenue, Cincinnati, Ohio 45229, USA; 4 Division of Human Genetics, Cincinnati Children's Hospital Medical Center, 3333 Burnet Avenue, Cincinnati, Ohio 45229, USA; 5 Division of Pediatric Urology, Cincinnati Children's Hospital Medical Center, 3333 Burnet Avenue, Cincinnati, Ohio 45229, USA; 6 Neurology, UC Physicians/ University of Cincinnati, 222 Piedmont Avenue, Ste. 3200, Cincinnati, Ohio 45219, USA

**Keywords:** Disorders of sex development, Ambiguous genitalia, 17-beta-HSD-3 deficiency, Gender assignment

## Abstract

**Background:**

17β-Hydroxysteroid dehydrogenase type-3 (17βHSD-3) deficiency is a rare cause of 46,XY disorders of sex development. The enzyme converts androstenedione to testosterone, necessary for masculinization of male genitalia in utero. 17βHSD-3 deficiency is frequently diagnosed late, at puberty, following virilization, with consequent female-to-male gender reassignment in 39-64%. The decision for sex of rearing is difficult, especially if diagnosed in early childhood. Consensus guidelines are equivocal or support male gender assignment. Long-term outcomes data to guide decisions are also lacking; however, in the few cases of early diagnosis and orchiectomy, female gender retention appears more likely.

We report two patients with 17βHSD-3 deficiency, who presented at unusual ages, in whom female gender was chosen. We performed a focused literature review and summary of gender outcomes in 17βHSD-3 deficiency following early orchiectomy.

**Cases:**

Patient A was a phenotypic female who presented at one year of age with bilateral inguinal hernias and external female genitalia. Testes were identified at surgery. The karyotype was 46,XY. She was initially diagnosed with complete androgen insensitivity syndrome; however, androgen receptor mutation analysis was negative. Human chorionic gonadotropin stimulation yielded a low testosterone: androstenedione ratio (0.6, normal >0.8). Genetic testing demonstrated compound heterozygosity for two known mutations of the *HSD17B3* gene. She underwent bilateral orchiectomy at two years of age.

Patient B was born with female genitalia and virilized at 13 years of age. She did not seek evaluation until 22 years of age. Her karyotype was 46,XY. She had bilateral inguinal testes and low testosterone: androstenedione ratio (0.3). *HSD17B3* gene sequencing showed her to be a compound heterozygote for two known mutations. She identified herself as female and underwent bilateral orchiectomy and estrogen replacement therapy.

**Conclusions:**

These two patients highlight the complexities of diagnosis and management in 17βHSD-3 deficiency. Although existing data are limited, early orchiectomy is likely to result in retention of female gender identity, avoiding the complications related to virilization in adolescence. As such, it is important to pursue a definitive diagnosis to guide clinical decisions, and to have the support and long term follow up with an inter-disciplinary disorders of sex development team.

## Background

Deficiency of the enzyme, 17β-hydroxysteroid dehydrogenase type 3 (17βHSD-3), is a rare autosomal recessive disorder of sex development (DSD) in individuals with 46,XY karyotype. 17βHSD-3 is found primarily in the testes and converts androstenedione to testosterone, which is necessary for normal masculinization of male external genitalia in utero. The majority of affected individuals are born with female or ambiguous external genitalia [1]. While some individuals may be diagnosed in infancy or early childhood after evaluation for ambiguous genitalia or inguinal hernia, many are not diagnosed until puberty, when they present with virilization. Virilization at puberty may be due to residual activity of 17βHSD-3 in the presence of increased androstenedione, as well as activity of extragonadal 17βHSD isoenzymes [2]. Gender reassignment from female to male has been reported in 39-64% in patients who were initially raised as girls [3].

While decisions regarding gender assignment in patients with 17βHSD-3 deficiency are difficult at any age, presentation in early childhood poses additional considerations, including the concern of performing orchiectomy before clear gender identity is evident and/or the patient is able to assent or consent for surgical procedures. Parents and clinicians must weigh the importance of deferring surgery until the affected individual is older, against irreversible surgery at a younger age which may or may not alter the child’s adjustment or parental distress [4,5]. However, in patients with 17βHSD-3 deficiency, waiting may not be optimal as a child raised as female will undergo virilization at puberty if the testes are retained, which will lead to its own set of medical and psychosocial implications. In addition, in patients who elect reassignment to male gender, masculinizing genitoplasty has less successful outcomes when done in adulthood [6].

Existing management guidelines are either equivocal or favor male gender assignment. The 2006 Chicago consensus statement recommends that the “combination of a male gender identity in the majority” but the “unknown” potential for fertility should be considered when assigning gender in affected infants [7]. The University of Oklahoma Health Sciences Center’s SUCCEED Clinic favors male gender assignment in infants with 17βHSD-3 deficiency as there is a high likelihood of male gender development. However, due to “an intermediate risk for germ-cell tumors” and “no reports of fertility thus far”, they state that “female gender assignment may be appropriate in some situations” [8]. Texas Children’s Hospital Multidisciplinary Gender Medicine Team guidelines recommend that “when the components of biological sex” (genomic, anatomic, hormonal, and brain sex) “…do not strongly align, the goals in such cases should be to preserve the anatomic and physiologic components of a change in gender identity later in a child’s life” [9]. Furthermore, long term outcomes data are lacking for patients with 17βHSD-3 deficiency, especially for those diagnosed at a young age. However, there are a small number of reported cases who were diagnosed in early childhood and retained female gender identity following orchiectomy [10-12].

We report two patients with 17βHSD-3 deficiency who presented at unusual ages, the first in early childhood (who underwent early orchiectomy) and the second in early adulthood. These patients highlight the difficulties encountered with diagnosis and management in this condition. They also uniquely frame the question of gender identity at opposite ends of the pediatric age spectrum, as female gender was maintained in both cases. We performed a focused review and simple tabulated summary of the existing literature to help guide counseling regarding gender outcomes with or without early orchiectomy.

## Case presentations

### Patient A

Patient A was a Caucasian female who presented to our center in early childhood. She was born with female external genitalia. When she was 16 months old, she underwent elective surgery for repair of umbilical and bilateral inguinal hernias. She was found to have bilateral inguinal gonads with testicular morphology. Laparoscopic evaluation demonstrated bilateral vas deferens and spermatic vessels; no uterus or ovaries were identified. Endoscopy of the vagina demonstrated a blind-ending vagina. Histopathology of the gonadal biopsy confirmed immature testicular tissue. Chromosome analysis revealed a 46,XY karyotype in all cells. She was initially thought to have complete androgen insensitivity syndrome (CAIS); however, sequencing of the androgen receptor gene was negative on two separate blood samples. Subsequent human chorionic gonadotropin (HCG) stimulation revealed a hormonal profile consistent with 17βHSD-3 deficiency: low stimulated testosterone to androstenedione (T/A) ratio of 0.6 (normal >0.8), and subnormal testosterone response of 2076 pmol/L (Table 1). Genetic sequencing of the *HSD17B3* gene demonstrated that she was a compound heterozygote for two known mutations: exon 3 (p.Arg80Trp) and exon 3/ intron 3 junction (c.325+4A->T).

**Table 1:** Hormonal profiles of patients A and B

	**Patient A**		**Patient B**	**Norms for pubertal males**
	**Baseline**	**Post HCG**	**Baseline**	
Androstenedione (A) (pmol/L)	768	3525	20242	1152 – 6700
Testosterone (T) (pmol/L)	796	2076	5363	12100 – 33560
Dihydrotestosterone (DHT) (pmol/L)	106	303	963	825 – 2235
T/A ratio		0.6	0.3	>0.8
T/DHT ratio		6.8	5.5	<20
Luteinizing hormone (IU/L)	3.8		26.8	3.0 – 10.5
Follicle stimulating hormone (IU/L)	1.7		29.0	4.8 – 20.0

After discussions regarding diagnostic implications and management options with our inter-disciplinary DSD team, Patient A’s parents chose to continue to raise their child as a female, and elected early orchiectomy to prevent virilization and eliminate any malignancy risk. Bilateral orchiectomy was performed at age 2.5 years. Histopathology confirmed normal testes containing predominantly Sertoli cells with a normal distribution of non-proliferating primitive germ cells. There was no evidence of malignancy. She is currently doing well at age 4 years, and there have been no concerns for gender dysphoria to date. She will receive estrogen replacement for puberty induction and long term hormonal therapy in the future.

### Patient B

Patient B was a Caucasian female who presented to our center in early adulthood. She was born a phenotypic female and was raised accordingly. At 13 years of age, she developed facial and body hair, deepened voice, and clitoromegaly. She suffered depression and anxiety regarding these physical changes, but did not seek medical evaluation until age 22 years, after she was hospitalized for depressive symptoms with suicidal ideation and diagnosed with bipolar disorder.

Physical examination on presentation was notable for facial hirsutism, generous bilateral labia majora, and no palpable inguinal or labial masses. The clitoris was 5 cm in length, but did not protrude beyond the labia. There was no vaginal introitus or dimple. The perianal area was normal. Magnetic resonance imaging of the pelvis demonstrated utero-vaginal agenesis, seminal vesicles at the base of the bladder, and bilateral inguinal gonads (right gonad 2.7 cm × 1.9 cm, left gonad 3.0 cm × 1.8 cm) with testicular morphology. Further workup revealed a 46,XY karyotype and a hormonal profile consistent with 17βHSD-3 deficiency: low T/A ratio (0.3, normal >0.8), and low testosterone concentration of 5363 pmol/L (Table 1). Genetic sequencing of the *HSD17B3* gene demonstrated that she was a compound heterozygote for two known missense mutations: exon 3 (p.Arg80Gln) and exon 9 (p.Val205Glu).

Patient B underwent psychological assessment and counseling. She identified herself as female, and chose to proceed with bilateral orchiectomy and estrogen replacement. Histopathology of the gonads confirmed the presence of testicular tissue with rete testis, epididymis, vas deferens, Leydig cell hyperplasia, but absent germ cells. There was no evidence of malignancy. Postoperatively, she was started on transdermal estrogen replacement, and instructed on techniques for progressive perineal dilation for vaginal creation. At age 28 years, she is currently doing well psychologically, and reports comfortable and satisfying vaginal intercourse. However, she continues to have significant hirsutism despite prior orchiectomy and good compliance with estrogen therapy.

## Discussion

These cases highlight the challenges involved with and lessons learned from the diagnosis and management of 17βHSD-3 deficiency. Patient A illustrates that 17βHSD-3 deficiency can be missed if the correct testing is not performed. Patients with 17βHSD-3 deficiency present with a primarily female phenotype similar to CAIS. Inappropriate diagnosis of CAIS may result in the recommendation to retain the gonads for later development of female secondary sexual characteristics, but will instead result in unwanted virilization. Therefore, it is important that 17βHSD-3 deficiency be considered in any 46,XY phenotypic female and that definitive diagnosis is pursued. This is critical for management, including gender assignment and the decisions related to orchiectomy.

The complexities of gender decisions are also highlighted by our patients’ disparate ages of diagnosis. Patients who are older, such as Patient B, have the ability to make their own healthcare decisions, including consent for surgical procedures. Patient B maintained female gender, raising the question of whether intervention with early orchiectomy to prevent virilization would have resulted in a better transition through puberty and reduced her psychological distress. Patient A presented an additional dilemma as she was diagnosed in early childhood, before an age when she could express her gender identity and assent to orchiectomy. In counseling Patient A’s parents, we considered the possibility that raising a child in a gender inconsistent with gonadal and chromosomal sex could lead to gender dysphoria. Presence of prenatal androgen production or a Y chromosome does not seem to reliably predict development of male gender identity [22,23]. Furthermore, it is unclear how low levels of androgen produced in individuals with 46,XY disorders of testosterone biosynthesis impact brain sex development. This unique patient subset may be similar to CAIS patients before puberty, lacking effective prenatal androgen exposure on the brain. Biological factors aside, socialization and learning have been shown to contribute significantly to gender identity and role development. The majority of individuals (approximately 75%) with 46,XY DSD are satisfied with their sex of rearing as either male or female, as determined by their parents and physicians [24]. Gender role also appears to develop in keeping with initial assigned sex as these individuals proceed through puberty and into adulthood [25].

Given the uncertainties involved in how best to manage a young child with 17βHSD-3 deficiency, all management options were presented to the parents of Patient A, including: (1) maintaining female gender with early orchiectomy, (2) delaying surgery to enable longitudinal gender assessment, with or without delaying puberty onset with leuprolide therapy, or (3) male gender re-assignment with early or late genital reconstruction. Patient A was already being raised as a girl, and her parents decided to proceed with early orchiectomy to maintain female gender.

In order for Patient A’s family to make an informed decision, we performed a focused review of the literature on gender outcomes in 17βHSD-3 deficiency (Table 2). Only patients initially raised female with follow-up to age 12 years or older and with documented gender identity were included. In addition, we assessed and summarized the influence of early orchiectomy (by age 10 years) on gender (Table 3), as this had not been collectively reported in the past. For the eight patients who had early orchiectomy, the majority underwent surgery in early childhood, with a single patient having surgery at age 10 years, prior to virilization. All eight subjects retained female gender identity after age 12 years, with the longest follow-up extending to age 26 years. One subject was assessed to have androgynous psychosexual development but did not reassign to male gender [10]. Of the remaining patients who did not undergo early orchiectomy, diagnosis occurred at or after puberty, following referral for virilization, lack of breast development, or primary amenorrhea. About half reassigned to male gender during adolescence or early adulthood, while half remained female, like Patient B. Of the case reports that described outcomes for those who remained female, orchiectomy was subsequently performed in adolescence or early adulthood (Table 2). These data suggest that there is insufficient evidence to confidently support male gender reassignment in the setting of early childhood diagnosis. Early orchiectomy may be justified in view of retention of female gender identity, but the data are limited.

**Table 2:** Gender outcomes in patients with 17βHSD-3 deficiency initially raised female

		**Female gender maintained**			**Female to male reassignment**	
**Study**	**Subjects (n)**	**Early orchiectomy (n)**	**No early orchiectomy (n)**	**Age of late orchiectomy (years)**	n*	**Age of reassignment (years)**
Omrani [13]	3		3	?	0	
Ismail [10]	13	5	2	?	6^ǂ^	12,14,14,19
Bertelloni [11]	2	1	1	12	0	
Mains [14]	1		1	15	0	
Lee [15]	4		4	12,14,14	0	
Mendonca [12]	10	2	5	15–34	3	15,15, 26
Rosler [16]	9		2	?	7	10, 12–18
Imperato [17]	1				1	>15
Millan [18]	1		1	?	0	
Lanes [19]	3				3	11–13
Imperato [20]	1				1	14
Akesode [21]	1				1	
Total	49	8	19		22	

Only subjects followed until at least 12 or more years of age, with explicitly stated gender identity, were reported. Early orchiectomy was defined as orchiectomy occurring by age 10 years.

*None of the patients who had female to male gender reassignment had had a history of early orchiectomy.

^ǂ^Two of the patients were assessed to have masculine psychosexual development, but their parents refused gender change.

**Table 3:** Influence of early orchiectomy on gender outcome in patients with 17βHSD-3 deficiency initially raised female

**Gender identity outcome**	**Early orchiectomy (n=8)**	**No early orchiectomy (n=41)**	**All patients (n=49)**
Male	0	22	22
Female	8	19	27
% with development of male gender identity	0	54	45

We recognize the limitations of presenting two patients in order to suggest recommendations regarding orchiectomy and sex of rearing. It is important to note that male genital reconstruction can be very difficult in these cases and, despite the degree of virilization seen with Patient B, the clitoris was quite small. This raises the concern that even after assignment to a male gender, there may be the potential for dissatisfaction with the cosmetic appearance of the genitalia [6]. To date, many of the outcomes regarding masculinizing surgery are either short term or retrospective, and may be based on outdated surgical practices.

More outcomes research is needed to guide gender decisions in patients diagnosed with this condition during early childhood. For patients raised female who undergo early orchiectomy, more long term outcomes data regarding gender identity is needed, as well as data related to quality of life, compliance with sex hormone replacement in adolescents and young adults, and sexual function, in order to better counsel families like those of Patient A. For patients raised male with retention of testes, more data regarding outcomes of masculinizing genitoplasty, sexual function, fertility, and malignancy risk is needed. Fertility in 17βHSD-3 deficiency does not appear likely. While there is a single reported case of a patient (with a history of masculinizing genitoplasty in adolescence) having fathered a child [6], documented testicular histology in these patients does not appear to be consistent with reproductive capacity. Gonadal tissue removed from younger patients demonstrates germ cells; however germ cells are absent, as in Patient B, or very immature at puberty [6,15]. It is unknown whether early testosterone therapy and orchidopexy would result in a more favorable outcome. With regards to malignancy, an intermediate risk (28%) of germ cell malignancy has been cited in those who did not undergo orchiectomy; however, this risk is based on only a few patients [7].

## Conclusions

Our two patients provide a unique opportunity to learn from opposite ends of the pediatric age spectrum. They highlight the complexities of diagnosis and management of this condition. Our summary of the literature on gender outcomes in 17βHSD-3 deficiency suggests that early orchiectomy is likely to result in retention of female gender identity. However, the existing data are still very limited, and there is a need for long term outcomes data in more patients, which may be best facilitated by national and international collaborative patient registries, given the rarity of this disorder. From a clinical perspective, pursuing a definitive diagnosis is critical, and patients and families need long term follow-up and support with an inter-disciplinary DSD team.

## Consent

Written informed consent was obtained from Patient A’s parents and Patient B for publication of this case report. Copies of the written consents are available for review by the Editor-in-Chief of this journal.

## Abbreviations

17βHSD-3: 17β-hydroxysteroid dehydrogenase type 3; DSD: Disorder of sex development; CAIS: Complete androgen insensitivity syndrome; HCG: Human chorionic gonadotropin; T: Testosterone; A: Androstenedione; DHT: Dihydrotestosterone.

## Competing interests

None of the authors have any competing interests to report.

## Authors’ contributions

All authors have made substantive intellectual contributions to this manuscript. JC and MR were responsible for conceptualizing, designing, writing and revising the report. JC did the background research. AV, LB, HS, SA and PC wrote sections of and revised the manuscript critically. All authors, individually and as a team, were directly involved in the clinical diagnosis and management of one or both patients. All authors have read and approved the final manuscript.
